# The Effects of Electric Field Dynamics on the Quality of Large-Area Nanofibrous Layers

**DOI:** 10.3390/polym13121968

**Published:** 2021-06-14

**Authors:** Marek Pokorný, Jan Klemeš, Adéla Kotzianová, Martin Fogl, Anna Zítková, Simon Jantač, Kateřina Knotková, Juraj Košek, Vladimír Velebný

**Affiliations:** 1 R&D Department, Contipro a.s., 56102 Dolní Dobrouč, Czech Republic; jan.klemes@contipro.com (J.K.); Martin.Fogl@contipro.com (M.F.); katerina.knotkova@contipro.com (K.K.); Vladimir.Velebny@contipro.com (V.V.); 2 Department of Chemical Engineering, University of Chemistry and Technology, Technická 5, 16628 Praha 6, Czech Republic; Anna.Zitkova@vscht.cz (A.Z.); simon.jantac@vscht.cz (S.J.); Juraj.Kosek@vscht.cz (J.K.)

**Keywords:** electrospinning, nanofibers, homogeneity, large-area production

## Abstract

This paper presents technological modifications of an electrostatic spinning device, which significantly increase the thickness homogeneity (i.e., quality) of produced layers by creating auxiliary dynamic electric fields in the vicinity of the spinning and collector electrodes. A moving body was installed above the needleless spinning electrode, which destabilized the standing wave occurring on the free surface of the spinning solution. Furthermore, an endless belt design was used for the collector electrode instead of a roll-to-roll design, which made it possible to substantially increase the surface speed of the substrate and, therefore, the dynamics of the electric field at the place of collection of the fibers being spun. As a result, the coefficient of variation of the area weight of 912 samples cut out from the deposited nanofibrous layer, which was (1000 × 500) mm^2^ in size and had an average area weight of (17.2 ± 0.8) g/m^2^, was less than 4.5%. These results were obtained only when the dynamics of both the spinning and collector electrodes were increased at the same time. These modifications resulted in a significant increase in the quality of deposited nanofibrous layers up to the standard required for their use in pharmaceutical applications.

## 1. Introduction

In the process of electrostatic spinning, nanofibers are randomly deposited onto the surface of a collector electrode, where they form a thin layer. The area distribution of deposited nanofibers and, therefore, the resulting thickness homogeneity of the nanofibrous layer are significantly affected by the distribution of static surface charges induced in the electrostatic field on the surface of the collector. This distribution of charges is influenced by the geometric parameters of the electrodes [1,2], local differences in the conductivity of the collector electrode surface [3], properties of the substrate material [4], distribution of the electrostatic field [5], properties of the already deposited nanofibrous layer, deposition time, and potentially other factors. During the deposition process, these local charges gradually acquire different intensities, new ones can occur in different locations, and existing ones can dissipate. The positions of these surface charges and the amount of time they act on the fibers being deposited cannot be independently controlled or monitored during the spinning process. The distribution of these charges affects the resulting thickness distribution across the deposited nanofibrous layer, and deviations in its homogeneity may reach 10 s of percent [6]. Whenever a large-area nanofibrous layer with a uniform thickness is being prepared, the effects of these surface charges prove to be a major issue.

Nanofibrous products have to comply with clearly defined requirements regarding their quality, one of which is the admissible deviation in the weight of each unit of the product. For example, in the case of pharmaceutical forms (tablet, capsule or powder) with an average unit weight of less than 80 mg, this deviation can be at most ±10% of the average weight of the given unit; for more information, see [7]. This could also be applied to other fields such as cosmetics or filtration. In laboratory and especially the large-scale production of nanofibrous layers, maintaining the deviation within this limit is still problematic; the products have to be individually checked (i.e., weighed) and carefully sorted. Thickness and therefore weight inhomogeneities in layers produced by electrospinning can have several different causes, as described in our previous study [8]. These laboratory results have also shown that the highest quality of deposited nanofibrous layers can be achieved by increasing the dynamics of the electric fields by setting the spinning nozzle and the surface of the substrate on the collector in motion. However, the application of these technical solutions in large-scale production of high-quality large-area nanofibrous layers poses considerable problems. Most of the production equipment available on the market uses a roll-to-roll system, where the dynamics of the electric field are influenced only by the movement of the substrate in the unwinding direction [9,10,11]. In our case, nanofibrous layers were produced using the unique production equipment, which enables us to increase the dynamics of the electric field, and we were able to relate these changes with the homogeneity of prepared nanofibrous layers. We focused on the production of nanofibrous layers with the intended use in cosmetics, where the large-area nanofibrous layers are cut to many products after its production and each product must meet quality requirements.

## 2. Hypothesis

**Figure 1:**
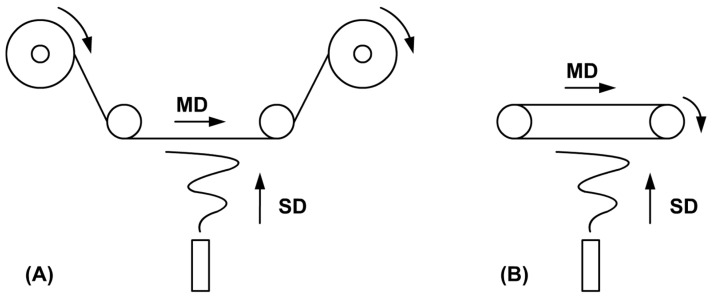
Diagrams of two fundamentally different basic collector designs with moving substrates used in industrial devices for electrostatic spinning—roll-to-roll (**A**) and endless belt (**B**), where SD is spinning direction and MD is machine direction.

One of the possible ways to mitigate or eliminate the negative effects of surface charges on the collector electrode and improve the quality of the produced nanofibrous layer is to set the charges in motion—to create a non-stationary electric field. A dynamic electric field can be created in the vicinity of the collector electrode by moving the surface of the collector electrode or by moving the substrate itself. Setting and keeping larger electrodes in motion is problematic from a technical point of view, which is why we focused on moving the substrate. The motion of the substrate depends on the technology used for its unwinding. Most of the devices that allow the preparation of large-area layers use winding and unwinding units with a roll-to-roll design [8]—see Figure 1A.

If we want to produce a layer with an area weight of *AW_RR_* (kg·m^2^) on a roll of substrate with a width of *SW* (m), we will need to unwind the roll at a speed of *SS* (m·s^−1^) based on the known production rate of nanofibers using the chosen nozzles of *PR* (kg·h^−1^). Therefore, we can say that the area weight *AW_RR_* is inversely proportional to the unwinding speed of substrate *SS* according to Equation (1).

(1) $$AW_{RR}=\frac{PR}{SW\cdot SS}$$

The speed of substrate is limited because the production rate is limited by the type of spinning nozzle being used and the applied voltage. For example, if our aim is to produce a layer with an area weight of *AW* = 0.01 kg·m^−2^ on a substrate with a width of *SW* = 1 m using a device with a maximum production rate of *PR* = 0.01 kg·h^−1^ and a roll-to-roll winding/unwinding unit, we have to set the unwinding speed of the substrate to *SS* = 1 m·h^−1^. It clearly follows that the dynamics of the electric field are limited by these process parameters and cannot be substantially increased. For this reason, it is better to use an unwinding unit with a substrate of a finite length looped into an endless belt—see Figure 1B. A different equation has to be used for this design to describe the relationship between the desired area weight *AW_EB_* and the individual parameters, as demonstrated by Equation (2).

(2) $$AW_{EB}=\frac{PR\cdot DT}{SW\cdot SL}$$

where *DT* (h) is the deposition time and *SL* (m) is the total length of the substrate. It follows from this equation that the resulting *AW* is directly proportional to the deposition time. Since the speed of substrate *SS* is not included in Equation (2), there is theoretically no limit to the value it can be set to. Therefore, this design makes it possible to significantly change the dynamics of the electric field in the vicinity of the collector electrode, and it can be assumed that there is a threshold speed, past which the effects of surface charges on the thickness homogeneity of deposited layers are reduced.

The substrate always moves in a single direction, which is called the machine direction (MD); its movement speed in this direction is equal to the value of *SS*. The direction perpendicular to the MD at the plane of the substrate surface is called the cross direction (CD). The substrate does not move in this direction at all, and the electric field acting in this direction can, therefore, be considered stationary as well. According to our hypothesis, movement and a dynamic electric field acting in this direction as well need to be introduced into the system. Furthermore, according to [12,13], a standing wave occurs on the free surface of a liquid in an electrostatic field, due to which fibers form in fixed positions at regular intervals, which depend on the intensity of the acting electric field.

One of the aims of this work was to confirm the hypothesis that this standing wave needs to be disrupted in a suitable way to ensure that fibers are deposited at short time intervals from varying places and no preferred or fixed positions, otherwise localized thicker deposits can form on the substrate in the CD, resulting in uneven layer thickness. In other words, variation over time needs to be introduced into the electric field in the CD as well in order to increase the degree of variation in the distribution of local surface charges on the substrate, which will, in turn, increase the thickness uniformity of the deposited nanofibrous layers.

The thickness homogeneity of a deposited nanofibrous layer can also be significantly affected by the evenness of the flow of the spinning solution from needleless nozzles, especially since they are several tens of centimeters long. The effects of the design of the distribution chambers inside the nozzle on the ejection of the spinning solution from the individual openings were confirmed with numerical simulations.

The thickness homogeneity of a nanofibrous layer can be evaluated using several different methods. One method suitable for the evaluation of the thickness homogeneity of low-weight nanofibrous layers that cannot be removed from the substrate in one piece consists of analyzing the intensity of light passing through such a layer, or more precisely, in analyzing its absorption by the nanofibrous layer manifested in lower pixel intensities of its images in places of greater thickness [14]. This method allows researchers to quickly assess the thickness distribution across the layer; however, different pixel intensities are not correlated with local differences in area weight. The obtained information needs to be subsequently verified using another method—by directly weighing individual parts of the analyzed layer. This method provides accurate information on differences in weight, which can be used to calculate the area weight of the analyzed nanofibrous layer. The drawback of this method is that it can be used only with self-supporting nanofibrous layers (removed from the substrate) and that it is destructive.

Based on the hypothesis described above, the electrospinning device was modified in two ways: (a) the endless belt design was used to move the substrate; (b) a moving body was installed immediately above the spinning electrode.

## 3. Materials and Methods

**Figure 2:**
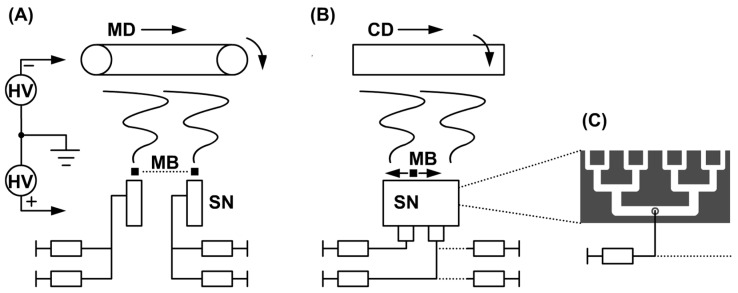
A diagram of the spinning system in the deposition chamber of the industrial device with two needleless spinning nozzles (SN) and a collector electrode with the endless belt design—shown from the cross direction (CD) (**A**) and machine direction (MD) (**B**). MB is moving body and HV is high voltage source. The third image shows the design of distribution chambers inside the needleless spinning nozzle (**C**); there were four of these segments in each nozzle.

An aqueous solution of a 6 wt % blend of hyaluronic acid (83 kDa, Contipro a.s., Dolní Dobrouč, Czech Republic) and polyethylene oxide (PEO, 600 kDa, Meisei Chemical Works, Ltd., Kyoto, Japan) combined at a ratio of 80:20 wt % was used for the electrospinning process. Altogether, six samples were prepared using an in-house assembled electrospinning pilot line. The solution was dosed at a rate of 2.0 mL·min^−1^ into two needleless spinning nozzles forming the spinning electrode with their longer sides aligned with the CD—see Figure 2A,B.

The outlets of the spinning nozzles were 550 mm long and the nozzles were 400 mm apart (measured in the machine direction). The flow of the spinning solution from the individual distribution chambers along the entire length of the spinning nozzle was studied using a computational fluid dynamics (CFD) simulation performed in the OpenFoam environment (OpenCFD Ltd., London, UK). The simulation was performed for one of the four separate parts of the spinning nozzle—see Figure 2C. The simulation took into account influencing parameters, such as the flow behavior of the liquid (viscosity, non-Newtonian fluid characteristics) and pressure losses in narrow chambers, i.e., capillary and surface forces. A more detailed description of the numerical simulation methods and parameters is described in the Supplementary Materials of this paper (Tables S1 and S2).

A moving body made of an electrically non-conductive material was installed (3 ± 1) mm above the upper edges of both spinning nozzles. This body could move in the CD along the entire length of the outlets of the spinning nozzles and during the entire spinning process. An electric potential of +45 kV was applied to the spinning nozzles. The solution was spun in an air-conditioned chamber at a relative humidity of (20 ± 5)% and a temperature of (23 ± 2) °C. Fibers were deposited onto the surface of a textile substrate belt located at a distance of 18 cm from the spinning nozzles. The substrate belt was attached to an electrically conductive foil, which served as a negatively charged electrode. An electric potential of −30 kV was applied to the foil. Both materials were looped into a single 120-cm-long endless belt. The morphology of nanofibrous samples was studied using electron microscopy (Carl Zeiss, Ultra Plus, Oberkochen, Germany). To prepare samples for the SEM analysis, the nanofibrous mats were coated with a very thin layer of Au/Pd (80/20) in a Leica EM ACE600 coater (Leica, Wetzlar, Germany). Images were acquired using an InLens SE detector (Carl Zeiss, Ultra Plus, Oberkochen, Germany), the working distance ranged from 2.6 mm to 4.0 mm, and the pressure was approximately 10^−4^ Pa.

To estimate the relationship between thickness homogeneity and the speeds of the belt and the moving body, we spun the first five samples over only 20 min but at various speeds. The speed of the belt was set to 25, 100 or 200 cm·min^−1^ in the MD and the speed of the moving body was set to 15 cm·s^−1^, or it remained static. Each sample was deposited three times using the same process parameters. Four photographs were taken at four different locations on each of the deposited layers in order to obtain relevant data. A total of 60 images were taken and analyzed, i.e., 12 images per sample. The thickness of these five samples of nanofibrous layers allowed us to photograph them against light using a Canon Coolpix P7700 camera (Canon, Tokyo, Japan). This method, although not as precise as some other ones, allowed us to quickly estimate the thickness homogeneity of these thin samples while they were still on the substrate. Ultimately, the deposition time was increased to 120 min and a nanofibrous layer (sample 6) with a higher area weight was prepared. The speed of the belt was set to 200 cm·min^−1^ and the speed of the moving body was set to 15 cm·s^−1^. The other parameters remained unchanged. The higher weight of the sample allowed us to remove it from the substrate and directly determine its weight. Thus, the thickness uniformity of this layer was not evaluated visually by analyzing its photograph but using a different, more precise method directly connected to the physical parameters of the sample. The produced (100 × 50) cm^2^ nanofibrous layer was cut into 912 round pieces with a diameter of 23 mm using a plotter (Zund, S3 M-800, Altstätten Switzerland). All the pieces were individually weighed using an analytical balance (Mettler Toledo, XSR205, Greifensee, Switzerland), and the weight of each piece was converted to area weight (per square meter).

## 4. Results and Discussions

**Figure 3:**
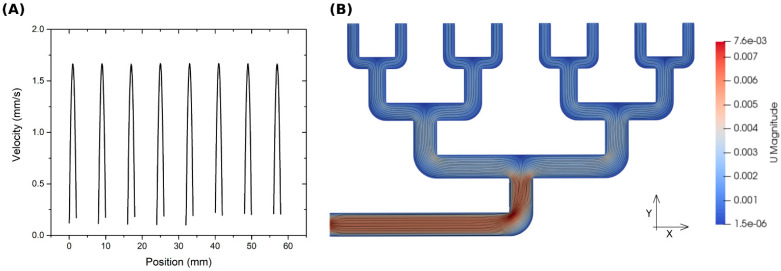
The result of a numerical simulation showing the speed (**A**) at which the spinning solution flowed out of the individual distribution chambers (**B**) in the needleless nozzles used in this study. One of four segments shown.

The results of the numerical simulation shown in the diagram in Figure 3 predicted that the liquid would flow at the same speed from all the distribution chambers. The results are shown for one of four identical segments, and we assumed the behavior would be identical along the entire length of the spinning nozzle.

**Figure 4:**
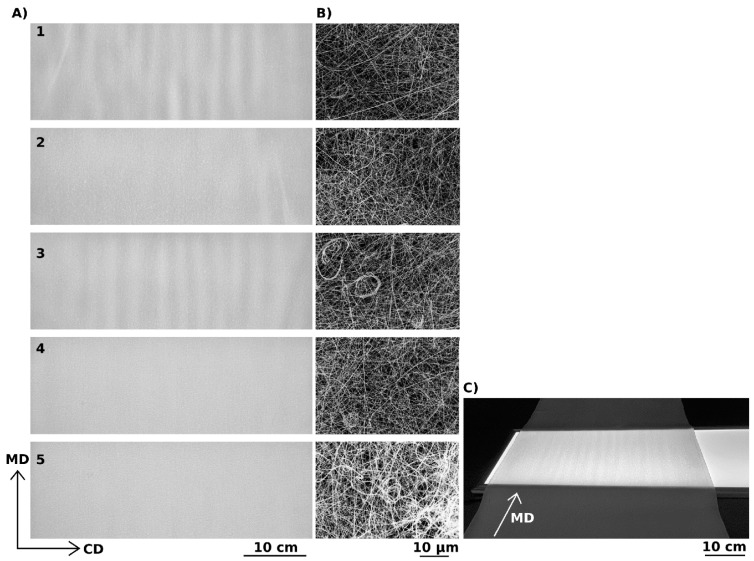
Images of nanofibrous layers photographed against light (**A**) and corresponding SEM images (**B**). An example of how the images were recorded in backlight (**C**). Each layer was prepared using different process parameters, see Table 1. CD is cross direction and MD is machine direction.

According to the results of the simulation, the effects of differences in the flow of the spinning solution from different parts of the nozzle on the uniformity of the deposited layer are negligible. Furthermore, we studied the effects of the movement of the body above the spinning electrode and of the speed of movement of the belt on the thickness uniformity of the deposited nanofibrous layers. These initial experiments were evaluated visually and using image analysis tools. The image analysis was based on the fact that the pixel intensity of an image of a deposited layer photographed against light corresponds with the thickness of the layer [14,15]. Figure 4 shows images of layers deposited at different movement speeds of the moving body above the spinning electrode and of the belt. The SEM analysis do not show significant differences in the nanofibrous structure—see Figure 4B.

**Figure 5:**
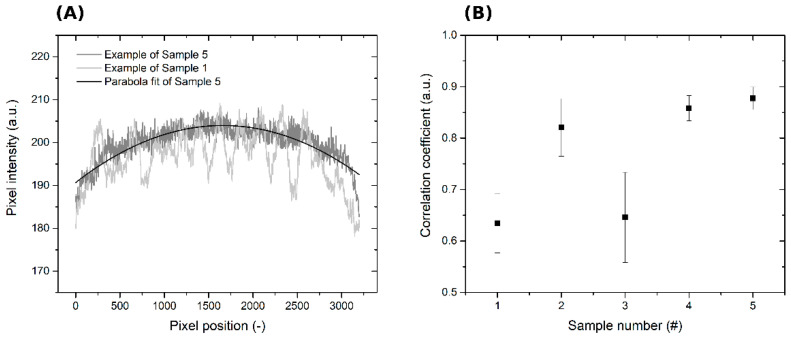
(**A**) Examples of waveforms of pixel intensities along a line in the CD taken from images of samples 1 and 5. A parabola fit of the sample 5 waveform. (**B**) Correlation coefficients of all the five samples.

The images in Figure 4A-1,A-3 clearly show bands in the structure of the deposited layers oriented in the MD, which formed when the body above the spinning nozzles was not activated or when the substrate moved too slow. A visual analysis of the images revealed that sample 5′s layers were the most homogeneous. These layers were produced when the moving body above the spinning nozzles was active and when the substrate moved at a speed of *SS* = 200 cm·min^−1^. For this reason, one waveform of variations in pixel intensity depending on their position along a line in the CD was selected from this series and fitted with a parabolic curve. The curve of the parabola is shallow, likely due to imperfections in the lighting panel (see Figure 4C) and the optical system of the camera used to photograph the layers. The coefficients of correlation between this curve and the waveforms of variations in pixel intensity depending on their position along a line in the CD were obtained for all five samples using data from all the taken images. The values of the correlation coefficients are directly proportional to the homogeneity of the nanofibrous layers. In addition, standard deviations of the individual average correlation coefficients were determined using all the images of the corresponding samples. These results are presented in Figure 5 and Table 1.

The results clearly show that in order to produce a nanofibrous layer with higher thickness homogeneity, the body above the spinning nozzles must be moving. Thickness homogeneity can be further increased by increasing the movement speed of the substrate to at least 100 cm·min^−1^. The results of the experiment confirmed the hypothesis described above and demonstrated that both modifications are required in order to obtain the best results. In other words, it is necessary to create dynamic electric fields in the vicinity of both electrodes at the same time, i.e., in the vicinity of the spinning nozzle and the collector.

**Figure 6:**
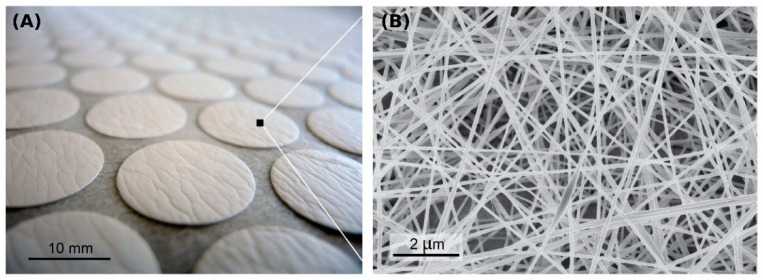
A photograph of the nanofibrous layer cut into 912 round pieces with a diameter of 23 mm (**A**). An electron microscope image of the structure of the nanofibrous layer (**B**).

Another experiment was performed to determine if the specified parameters would also be suitable for the preparation of a homogeneous layer with a higher area weight. The increased weight would allow us to evaluate the layer by directly weighing it instead of analyzing its images taken against light. For this purpose, the area weights of 912 pieces cut out from a deposited layer were analyzed. Figure 6A shows an image of the analyzed round pieces cut out from the layer, and Figure 6B shows an electron microscope image of the nanofibrous structure of the layer.

**Figure 7:**
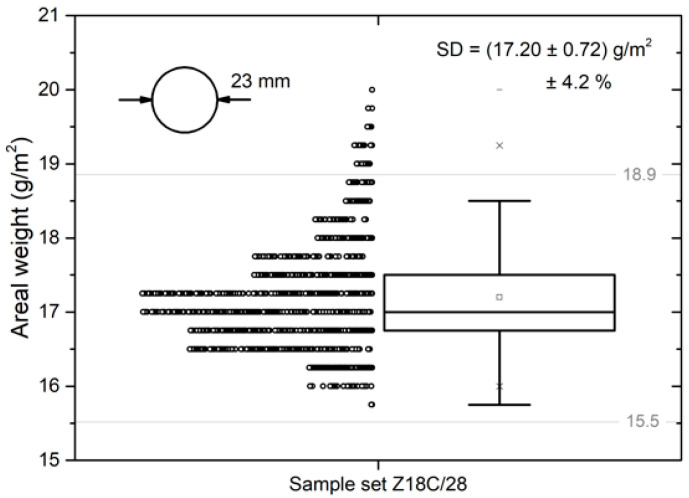
Area weights of all the cut-out pieces.

The statistical data obtained by calculating the area weights of the individual round pieces of the nanofibrous layer are provided in the chart in Figure 7. The average area weight of the pieces was *AW_EB_* = (17.20 ± 0.72) g·m^−2^. The coefficient of variation of the area weight of the evaluated pieces was 4.2%. A total of 879 pieces, i.e., 96.3% of all the pieces, had an area weight that was within the ±10% deviation range acceptable in pharmaceutical applications.

The pieces whose area weight was outside this range were taken from the edges of the deposited area, and in all cases, the samples had an area weight that was higher than intended. This result confirmed that the thickness of the deposited nanofibrous layer was highly uniform.

## 5. Conclusions

In this paper, we focused on the preparation of large-area nanofibrous layers with the aim of achieving uniform thickness across the entire deposited area. We hypothesized that thickness uniformity could be increased by creating dynamic electric fields in the vicinity of the spinning nozzles as well as the collector electrode. These electric fields have several consequences and they disrupt the effect of charge on fibers deposited on the substrate as well as patterns created around spinning electrodes. The results have shown that visible thickness inhomogeneities form in layers deposited using approximately static fields, which was confirmed by analyzing images of the layers photographed against light. The thickness uniformity of deposited layers increased significantly when dynamic electric fields were created simultaneously in the vicinity of the spinning nozzles by a moving body and in the vicinity of the collector electrode by the surface of the substrate moving at a high speed. An analysis of the produced layer showed that the area weights of 96.3% of all tested pieces were within the acceptable deviation range of ±10%; this was also true for the sample with a higher area weight. These results are very promising, and they indicate that this solution could allow the electrospinning of nanofibrous layers usable in pharmaceutical products.

## 6. Patents

Patent application PCT/CZ2019/050026 is resulting from the work reported here.

## Figures and Tables

**Table 1:** Results of the image analysis of layers deposited using different process parameters.

Sample Number	Speed of the Body Above the Nozzles in CD (cm·s^−1^)	Speed of the Belt in MD (cm·min^−1^)	Correlation Coefficient (a.u.)	Standard Deviation of the Correlation Coefficient (a.u.)
1	0	25	0.64	0.06
2	15	25	0.82	0.06
3	0	100	0.65	0.09
4	15	100	0.86	0.03
5	15	200	0.88	0.03

## Data Availability

The data presented in this study are available on request from the corresponding author.

## Supplementary Materials

The following are available online at https://www.mdpi.com/article/10.3390/polym13121968/s1, Table S1: NETGEN 2D Hypothesis Construction, Table S2: SimpleFoam boundary conditions.

## References

[1] Pokorný M., Klemeš J., Kotzianová A., Kohoutek T., Velebný V. (2017). Increased thickness uniformity of large-area nanofibrous layers by electrodynamic spinning. AIP Adv..

[2] Zhang D., Chang J. (2007). Patterning of Electrospun Fibers Using Electroconductive Templates. Adv. Mater..

[3] Dempsey D.K., Schwartz C.J., Ward R.S., Iyer A.V., Parakka J.P., Cosgriff-Hernandez E.M. (2010). Micropatterning of Electrospun Polyurethane Fibers Through Control of Surface Topography. Macromol. Mater. Eng..

[4] Collins G., Federici J., Imura Y., Catalani L.H. (2012). Charge generation, charge transport, and residual charge in the electrospinning of polymers: A review of issues and complications. J. Appl. Phys..

[5] Viswanadam G., Chase G.G. (2013). Modified electric fields to control the direction of electrospinning jets. Polymer.

[6] Senturk-Ozer S., Ward D., Gevgilili H., Kalyon D.M. (2012). Dynamics of electrospinning of poly(caprolactone) via a multi-nozzle spinneret connected to a twin screw extruder and properties of electrospun fibers. Polym. Eng. Sci..

[7] (2019). Uniformity of mass for single-dose preparation. The International Pharmacopoeia, 5.2.

[8] Yener F., Jirsak O. (2012). Comparison between the Needle and Roller Electrospinning of Polyvinylbutyral. J. Nanomater..

[9] Persano L., Camposeo A., Tekmen C., Pisignano D. (2013). Industrial upscaling of electrospinning and applications of polymer nanofibers: A review. Macromol. Mater. Eng..

[10] Müller F., Zainuddin S., Scheibel T. (2020). Roll-to-Roll Production of Spider Silk Nanofiber Nonwoven Meshes Using Centrifugal Electrospinning for Filtration. Appl. Mol..

[11] Song J., Liu Z., Li Z., Wu H. (2020). Continuous production and properties of mutil-level nanofiber air filters by blow spinning. RSC Adv..

[12] Lukas D., Sarkar A., Pokorny P. (2008). Self-organization of jets in electrospinning from free liquid surface: A generalized approach. J. Appl. Phys..

[13] Jiang G., Johnson L., Xie S. (2019). Investigations into the mechanisms of electrohydrodynamic instability in free surface electrospinning. Open Phys..

[14] Pokorný M., Kotzianová A., Rassushin V., Klemeš J., Velebný V. (2018). Rapid Visualization of the Mass Distribution Profile of Electrospun Layers. Acta Polytech..

[15] Kotzianova A., Klemes J., Zidek O., Mlynar Z., Pokorny M., Velebny V. (2019). Effect of different emitter types on the production of nanofibrous tubular structures: Thickness uniformity and productivity. AIP Adv..

