# Supplementary material for: The Effects of Electric Field Dynamics on the Quality of Large-Area Nanofibrous Layers
Source: Polymers (Basel). 2021 Jun 14;13(12):1968. doi: 10.3390/polym13121968 (PMC8232119; doi:10.3390/polym13121968)
Supplement: Supplementary file 1 [file polymers-13-01968-s001.zip › polymers-1239712-supplementary.pdf]

Supplementary Materials

The Effects of Electric Field Dynamics on the Quality of Large-Area Nanofibrous Layers

Marek Pokorný [1,*], Jan Klemeš [1], Adéla Kotzianová [1,*], Martin Fogl [1], Anna Zítková [2], Simon Jantač [2], Kateřina Knotková [1], Juraj Košek [2] and Vladimír Velebný [1]

[1] R&D Department, Contipro a.s., 56102 Dolní Dobrouč, Czech Republic; jan.klemes@contipro.com (J.K.); Martin.Fogl@contipro.com (M.F.); katerina.knotkova@contipro.com (K.K.); Vladimir.Velebny@contipro.com (V.V.)
[2] Department of Chemical Engineering, University of Chemistry and Technology, Technická 5, 16628 Praha 6, Czech Republic; Anna.Zitkova@vscht.cz (A.Z.); simon.jantac@vscht.cz (S.J.); Juraj.Kosek@vscht.cz (J.K.)
* Correspondence: marek.pokorny@contipro.com (M.P.); Adela.Kotzianova@contipro.com (A.K.)

Model goals and description

The OpenFoam environment was used to model the flow inside the distributor (Figure 2C). The goal of the model was to show that flow distributor design will grant a) the same exit velocity from all eight exit channels, b) the uniform initial filling of distributor, i.e., countering the effect of capillary forces causing the non-uniform filling, c) proper emitter work for the reasonable range of viscosities, surface tensions and inlet velocities with respect to the experimental conditions. To achieve this goal, two simulation methods were used.

The first method was the stationary simulation of incompressible flow using the Semi-Implicit Method for Pressure Linked Equations (SIMPLE) algorithm. The SIMPLE algorithm solves the continuity and Navier-Stokes equations eq (1, 2) (simpleFoam), where ρ is the density of solution, $\boldsymbol{v}$ is the velocity , ν is dynamic viscosity , and p is kinematic pressure (i.e. static pressure divided by the density of solution). The time derivative is zero because we are at a stationary state. This simulation was used to validate that the same outlet velocity profile, and the volumetric flow is achieved for each outlet during the spinning.

$$\nabla \cdot \boldsymbol{v} = 0 \tag{1}$$

$$\frac{\partial v}{\partial \mathrm{t}} + \nabla \cdot (\boldsymbol{v}\boldsymbol{v}) - \nu \Delta \boldsymbol{v} = -\nabla \mathrm{p} \tag{2}$$

The second type of simulation was used to prove the uniform distributor filling and the ability of distributor geometry to counteract the non-uniform filling possibly occurring due to the capillary forces. For this simulation, the combination of SIMPLE and PISO (Pressure Implicit with Splitting of Operator) algorithms with the addition of the Volume of Fluid (VoF) method were used (interFoam). In addition to continuity and Navier-Stokes equations (eq. 1,2) the equation of interphase is solved (eq3). The implementations of both used algorithms are in the default OpenFoam library.

$$\frac{\partial \alpha}{\partial t} + \frac{\partial (\alpha v_j)}{\partial x_j} = 0 \tag{3}$$

SALOME 9 meshing setup

The design of the distributor was imported to Salome 9 (https://www.salome-platform.org/) in the form of a CAD file (Step format). In Salome 9, the 2D mesh was created and preprocessed. The CAD model was cut with a plane. The resulting 2D geometry was then preprocessed, i.e. the groups for boundary conditions were set up, and the mesh was created and extruded by one cell, so the cells have physical volume (required procedure to calculate 2D geometry in the OpenFoam). The hypothesis for the meshing was NETGEN 2D, which produces the first order triangular mesh. The detailed setup is in Table 1.

Table 1.NETGEN 2D Hypothesis Construction

PROPERTY NAME	VALUE
MAX. SIZE	$5\cdot10^{-3}$,$5\cdot10^{-4}$,$2\cdot10^{-4}$,$1\cdot10^{-4}$ m
MIN. SIZE	0 m
FINENESS	Very fine
GROWTH RATE	0.1
NB. SEGMENTS PER EDGE	3
NB. SEGMENTS PER RADIUS	5
CHORDAL ERROR	-1
LIMIT SIZE BY SURFACE CURVATURE	True
QUAD-DOMINATED	False
SECOND ORDER	False
OPTIMIZE	True
ELEMENT SIZE WEIGHT (OPTIMIZER)	0.2
NB. SURFACE OPTIMIZIZATION STEPS (OPTIMIZER)	3

The size of elements was set to several values to test whether it influences simulation results or not. The total number of elements in the mesh were 1 431, 2 543, 17 953, 74 335 for maximal element sizes of $5\cdot10^{-3}$,$5\cdot10^{-4}$,$2\cdot10^{-4}$,$1\cdot10^{-4}$ mm respectively. Then the quality of mesh was evaluated, and the mesh was exported to the OpenFoam compatible format.

OpenFoam confguration

As the Reynolds number for the experimental ranges of inlet velocities were from 10^{-4} to 10^{-3}, the flow regime in the distributor is laminar. Therefore turbulent model was set to laminar. Although the solution has non-Newtonian behaviour, at a low shear rate occurring in the distributor, the non-Newtonian behaviour of solution does not occur, and the dynamic viscosity has a constant value of 1.5 Pa·s, which translates to the value of $1.6\cdot10^{-3}$ $m^2\cdot s^{-1}$ for kinematic viscosity for our solutions.

For VoF simulation, the kinematic viscosity of air was set to $1.48\cdot10^{-5}$ $m^2\cdot s^{-1}$, with the density of 1 $kg\cdot m^3$, the contact angle of steel-air-solution was measured to be 45.8°, and the same value was used in the model. The surface tension was set in the ranges of 20-70 $mN\cdot m^{-1,}$ which is believed to be the reasonable estimate of surface tensions for the used solutions. In the initial state of the simulations, the inlet boundary had the volumetric fraction of solution of 1. The rest of the mesh was empty (i.e., the volumetric fraction of solution is equal to 0). In this initial state, the model simulates the filling of the distributor (emitter). The configuration files fvSchemes and fvSolutions were kept at default values.

$$U(r) = U_{max}\left(1 - \frac{r}{R}\right)^2 \quad (4)$$

Table 2. Simple Foam boundary conditions

Kinematic pressure

PATCH NAME	TYPE OF BOUNDARY CONDITIONS
OUTLET	fixedValue (0)
WALL	zeroGradient
INLET	ZeroGradient
EMPTY	Empty

Velocity

PATCH NAME	TYPE OF BOUNDARY CONDITIONS
OUTLET	zeroGradient
WALL	noSlip
INLET	parabolic profile (see eq. 4)
EMPTY	Empty

InterFoam boundary conditions

Hydrostatic pressure

PATCH NAME	TYPE OF BOUNDARY CONDITIONS
OUTLET	fixedValue (0)
WALL	fixedFluxPressure
INLET	ZeroGradient
EMPTY	Empty

Velocity

PATCH NAME	TYPE OF BOUNDARY CONDITIONS
OUTLET	zeroGradient
WALL	noSlip
INLET	parabolic profile (see eq. 4)
EMPTY	Empty

Liquid phase

PATCH NAME	TYPE OF BOUNDARY CONDITIONS
OUTLET	zeroGradient
WALL	constantAngleContactAngle (45.8°)
INLET	inletOutlet (1)
EMPTY	Empty
